# Genetic assessment of inbred chicken lines indicates genomic signatures of resistance to Marek’s disease

**DOI:** 10.1186/s40104-018-0281-x

**Published:** 2018-09-13

**Authors:** Lingyang Xu, Yanghua He, Yi Ding, George E. Liu, Huanmin Zhang, Hans H. Cheng, Robert L. Taylor, Jiuzhou Song

**Affiliations:** 1 Department of Animal & Avian Sciences, University of Maryland, College Park, MD 20742 USA; 2 Animal Genomics and Improvement Laboratory, U.S. Department of Agriculture-Agricultural Research Services, Beltsville, MD 20705 USA; 3 Laboratory of Molecular Biology and Bovine Breeding, Institute of Animal Science, Chinese Academy of Agricultural Sciences, Beijing, 100193 China; 4 United States Department of Agriculture-Agriculture Research Service (ARS), Avian Disease and Oncology Laboratory, East Lansing, MI 48823 USA; 5 Animal and Nutritional Sciences, Davis College of Agriculture, West Virginia University, Morgantown, WV 26506 USA

**Keywords:** Genetic structure, Genomic signature, Homozygosity, Marek’s disease, Recombinant Congenic Strains (RCS)

## Abstract

**Background:**

Marek’s disease (MD) is a highly contagious pathogenic and oncogenic disease primarily affecting chickens. However, the mechanisms of genetic resistance for MD are complex and not fully understood. MD-resistant line 6_3_ and MD-susceptible line 7_2_ are two highly inbred progenitor lines of White Leghorn. Recombinant Congenic Strains (RCS) were developed from these two lines, which show varied susceptibility to MD.

**Results:**

We investigated genetic structure and genomic signatures across the genome, including the line 6_3_ and line 7_2_, six RCSs, and two reciprocally crossed flocks between the lines 6_3_ and 7_2_ (F1 6_3_ × 7_2_ and F1 7_2_ × 6_3_) using Affymetrix® Axiom® HD 600 K genotyping array. We observed 18 chickens from RCS lines were specifically clustered into resistance sub-groups distributed around line 6_3_. Additionally, homozygosity analysis was employed to explore potential genetic components related to MD resistance, while runs of homozygosity (ROH) are regions of the genome where the identical haplotypes are inherited from each parent. We found several genes including *SIK, SOX1, LIG4, SIK1* and *TNFSF13B* were contained in ROH region identified in resistant group (line 6_3_ and RCS), and these genes have been reported that are contribute to immunology and survival. Based on *F*_ST_ based population differential analysis, we also identified important genes related to cell death and anti-apoptosis, including *AKT1*, *API5*, *CDH13*, *CFDP* and *USP15*, which could be involved in divergent selection during inbreeding process.

**Conclusions:**

Our findings offer valuable insights for understanding the genetic mechanism of resistance to MD and the identified genes could be considered as candidate biomarkers in further evaluation.

**Electronic supplementary material:**

The online version of this article (10.1186/s40104-018-0281-x) contains supplementary material, which is available to authorized users.

## Background

Poultry products are main components of our daily life. The complexity of food safety and quality issues facing the poultry industry are rapidly escalating, and many other critical issues (i.e., bioterrorism, environmental stewardship and global competitiveness) are emerging simultaneously. Marek’s disease (MD) is a T cell lymphoma induced by the widespread and readily transmissible Marek’s disease virus (MDV) [1]. MD is one of the main chronic infectious diseases threatening the poultry worldwide, and interest for its economic importance to the commercial poultry industry.

MDV transforms mainly CD4+ T cells and causes various clinical syndromes in chicken tissues, which include peripheral nerves, gonad, iris, muscle, viscera, and skin [2]. MDV is shed and transmitted between birds via epithelial cells of the feather follicle, dander, chicken house dust, feces and saliva. Vaccines were developed to control the disease but are not sterilizing allowing the virus to replicate and spread. Thus, in the last few decades, field strains of the virus have evolved resulting in new subtypes that the vaccines may not completely control [3]. MDV has proven to be a valuable comparative biomedical model organism for understanding the principles of human disease [4] and MD is a natural model for lymphomas overexpressing Hodgkin’s disease antigen [5]. However, there is a shortage of genetic information about the virus host and etiological process leading to MDV uptake, dissemination, latency and tumor formation.

Currently, control strategies for MD predominantly rely on vaccination of chickens. But the vaccination cannot provide complete protection because of the changing nature of the disease itself and evolution of MDV with escalated virulence [4]. Recently, studies using integrating genomic approaches identified genes and molecular markers associated with MD disease resistance [6]. Genetic makers, quantitative trait loci (QTL) and genomics regions in a large reciprocal backcross (BC) were discovered between two partially inbred commercial White Leghorn layer chicken [7] and in an F6 advanced intercross population of commercial layer chickens [8]. Additionally, gene expression using transcript array [9, 10] and allele-specific expression analysis using RNA sequencing [11] have been utilized to understand response to MDV infection and MD genetic resistance.

To determine the genetic components pertaining to disease resistance, the Avian Disease and Oncology Laboratory (ADOL) has successfully developed inbred chicken lines resistant or susceptible to MD as a powerful experimental model for investigation of host resistance to this disease, the inbred lines 6_3_ and 7_2_. The percentage of chickens developing a tumor or tumors induced by a partially attenuated vv + strain of MDV differed significantly between the two lines of chickens. Genetic resistance to MD in chickens is commonly evaluated with MD incidence post-MDV challenge, which had been descripted in previous study [12]. Only 0–3% of line 6_3_ chickens developed tumors 8 wk post infection compared to 99%–100% of line 7_2_ chickens. Importantly, 19 recombinant congenic strains (RCS) have been developed using the inbred lines 6_3_ and 7_2_ as progenitor lines. The tumor incidence differs among RCS either vaccinated or unvaccinated. Thus, the varied resistance among the RCS can provide an ideal model to investigate genomic components that play vital roles in genetic resistance to MD. Although a previous study has demonstrated some genetic variability among the RCSs by microsatellite fingerprinting [2], genetic variations underlying varied susceptibility to MD in the RCS lines remains poorly understood.

In this study, we hypothesized that differential genomic signatures contribute to MD resistance in the RCS. Using ten lines, including line 6_3_ and line 7_2_, two hybrid F1 (F1 6_3_–7_2_ and F1 7_2_–6_3_) and six RCSs representing various capacity in resistance/susceptibility to MD, we investigated genetic structure in the chicken lines based on Affymetrix® Axiom® HD 600 K SNP array. Additionally, homozygosity analysis and genomic signatures were carried out to explore potential genetic mechanism of MD disease. Our studies revealed several genes related to regulation of cell death and anti-apoptosis, which are probably related to the resistance to MD.

## Methods

### Experimental population

In total, 30 chickens without treatments were selected and genotyped in this study. Among them, three were from each of the line 7_2_, line 6_3_, reciprocal cross F1 hybrid 7_2_ × 6_3_, F1 hybrid 6_3_ × 7_2_, and six recombinant congenic strains (RCSs) [C, J, M, N, S and X] [11]. RCSs were developed using line 6_3_ as the parental strain mated to line 7_2_ and then backcrossed to line 6_3_ twice followed by full-sib matings for about 20 generations. Eventually, diverse RCSs were generated and they contain 87.5% of line 6_3_ and 12.5% of line 7_2_ in the genetic background but with different MD resistance (Additional file 1: Figure S1) [3, 13]. In this study, six RCSs above were chosen to check their genetic background and reveal their particular resistant signatures.

### Genotyping and quality control

Genomic DNA from red blood cells was extracted using the DNeasy Blood & Tissue Mini Kit (QIAGEN). Genotyping for the 30 chickens was performed using the Affymetrix® Axiom® Genome-Wide Chicken Genotyping Array (600 K) by the Affymetrix service facility according to the manufacturer’s protocols [14]. Affymetrix adopts a preliminary call rate of 90% that is used by default to pass arrays for further data analysis. Successfully passed arrays were clustered and final genotypes were generated using the Axiom GT1 algorithm. Quality control was assessed in Genotyping Console v4.1.3. All chosen samples genotyped on Affymetrix SNP array met the 99% call rate.

### Multidimensional scaling and admixture analysis

Quality control were considered using the following selection criteria: MAF ≥ 0.01 (including SNPs with MAF ≥ 0.01), geno ≥0.1 (including only SNPs with a 90% genotyping rate or higher), and *P* value of *x*^2^ test for Hardy-Weinberg equilibrium ≥10^− 6^. To avoid linkage distortion for the population structure analysis, SNPs were pruned for linkage disequilibrium (LD) using pair-wise genotype correction (*r*^2^ > 0.2) in 50 SNP sliding widows with a step of 10 SNPs across the genome. To look for evidence of population substructure, we carried out Multidimensional Scaling (MDS) analysis using 5,064 LD filtered SNPs among all 30 chickens. Two dimensions in MDS analysis were calculated based on the identity-by-descent (IBD) pairwise distance among all chickens using PLINK, then MDS was plotted using the first-dimension values against the second-dimension values. To estimate the genetics admixture within 10 lines, STRUCTURE v2.3 was employed using the same LD filtered data set with 5,000 replicates and 2,000 burn-in cycles under the admixture model and correlated allele frequencies [15, 16].

### Phylogenetic analysis

Neighbor-joining (NJ) tree was constructed based on the estimation of IBD. As line 7_2_ shows significantly susceptible to MD compared to the RCS, we used line 7_2_ as the outgroup for NJ trees analysis. Pairwise genetic distance (D) between chickens was calculated using PLINK, where D = 1-[IBS2 + 0.5IBS1)/N]: IBS2 and IBS1 are the number of loci that share either 2 or 1 alleles identical by state (IBS), respectively, and the N is the number of loci [17, 18]. Then, phylogenetic tree was generated using FigTree 1.3.1 (http://tree.bio.ed.ac.uk/software/figtree/). In addition, Reynolds’ distance between pairwise inbred lines was estimated using Arlequin version 3.5 [19].

### Differential genomic signature

To investigate genome-wide patterns of genetic polymorphism related to susceptibility and resistance during the inbreeding process, the global *F*_ST_ was calculated for each single locus using Genepop software across all lines [20], which measured genetic differentiation between subpopulations using the approach previously described by Weir et al. [21]. Genomic signatures, implying divergent selection among inbred lines, can be recognized when *F*_ST_ values for the adjacent SNPs in a special window size were estimated to exceed the significant threshold [22]. We estimated average *F*_ST_ values of each nonoverlap region with 50 SNPs windows size. The top 1% regions were considered as candidate regions. Furthermore, we performed gene annotation based on genome assembly (Gallus_gallus-4.0) to identify the candidate genes involved in divergent selection during the inbred process.

### Runs of homozygosity

Runs of homozygosity (ROH) are regions of the genome where the identical haplotypes are inherited from each parent. The exploration of ROH can help to identify recessive disease variants and investigate the effects of genome-wide homozygosity on traits of biomedical importance. To investigate homozygosity distribution in six RCS lines as well as the parental line 6_3_ and line 7_2_, we performed ROH analysis with PLINK using a total of 527,021 SNPs [17]. A sliding window of 50 SNPs was used to identify ROH based on filtered SNPs. Here, homozygous segments were defined as more than 50 homozygous SNPs spanning larger than 500 kb. To avoid underestimation of ROHs caused by occasional genotyping error or missing genotype occurring in an otherwise-unbroken homozygous segment, we used the option --homozyg-window-het 1 --homozyg-window-missing 2 in PLINK to allow one heterozygous and two missing calls per window.

### Gene Ontology (GO) enrichment analysis

To assess the function of the positive selected genes identified in top 1% windows regions and genes in the ROH segments, we performed gene enrichment annotation and gene functional classification with DAVID (version 6.7) [23]. The default sets were used in DAVID with gene annotations based on *Gallus gallus*, and GOTERM_BP_FAT, GOTERM_CC_FAT, GOTERM_MF_FAT were selected as the functional annotation category.

## Results

### Summary statistics of SNPs array of QC

The quality of all SNPs in the array met the 99% call rate (Additional file 1: Figure S2). In the present study, a total of 527,021 SNPs in autosome chromosomes were kept for further analysis. Markers with high missing gencall rate (> 0.1), low MAF (< 0.01) and significant deviation from Hardy-Weinberg equilibrium (*P* < 1 × 10^− 6^) were excluded, leaving a total of 155,216 autosomal SNPs in population genetic structure analysis. Distributions in autosomes for total SNPs and filtered SNPs were presented in Additional file 1: Figures S3 and S4.

### Genetic structure and phylogenetic analysis

**Fig. 1:**
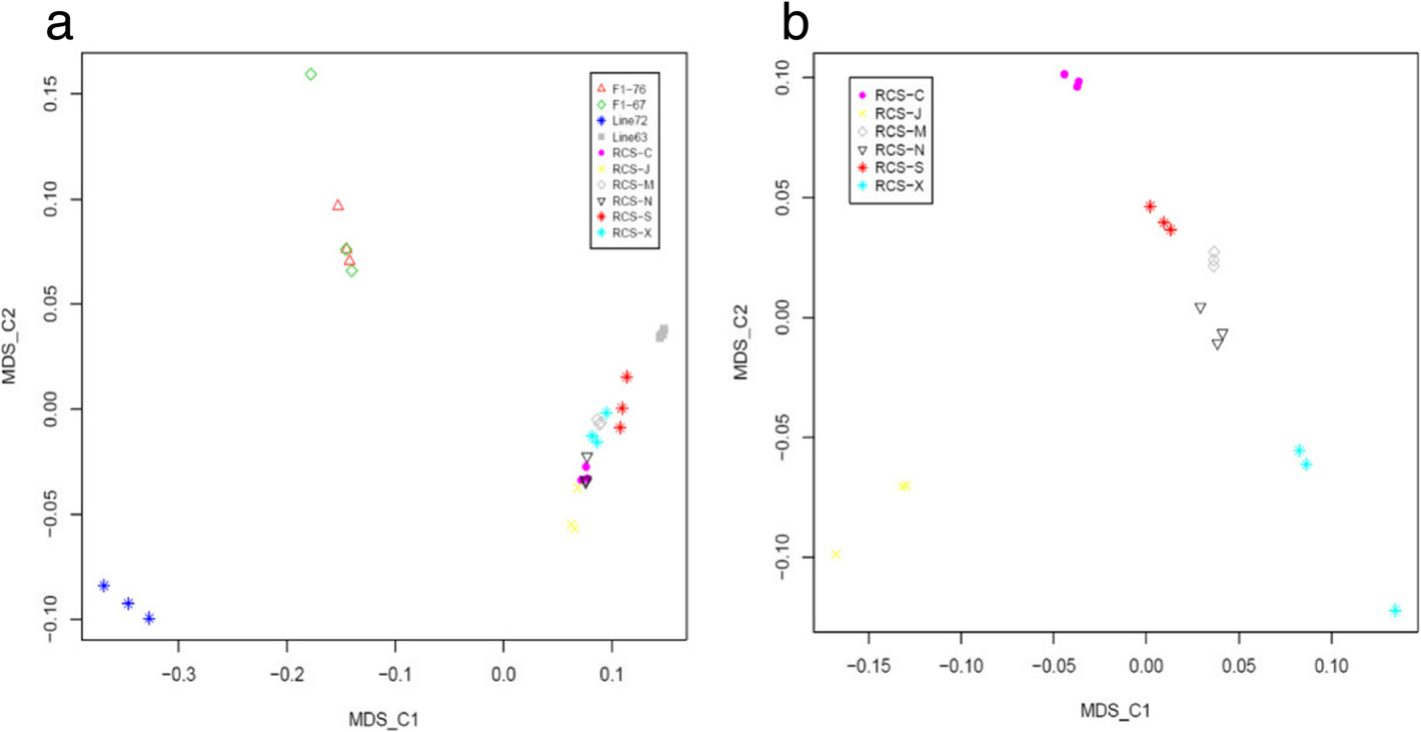
**a** Multidimensional scaling analysis (MDS) based on genome-wide IBS pairwise distances in a total of 30 chickens. **b** Multidimensional scaling analysis based on genome-wide IBS pairwise distances within 18 RCS chickens

**Fig. 2:**
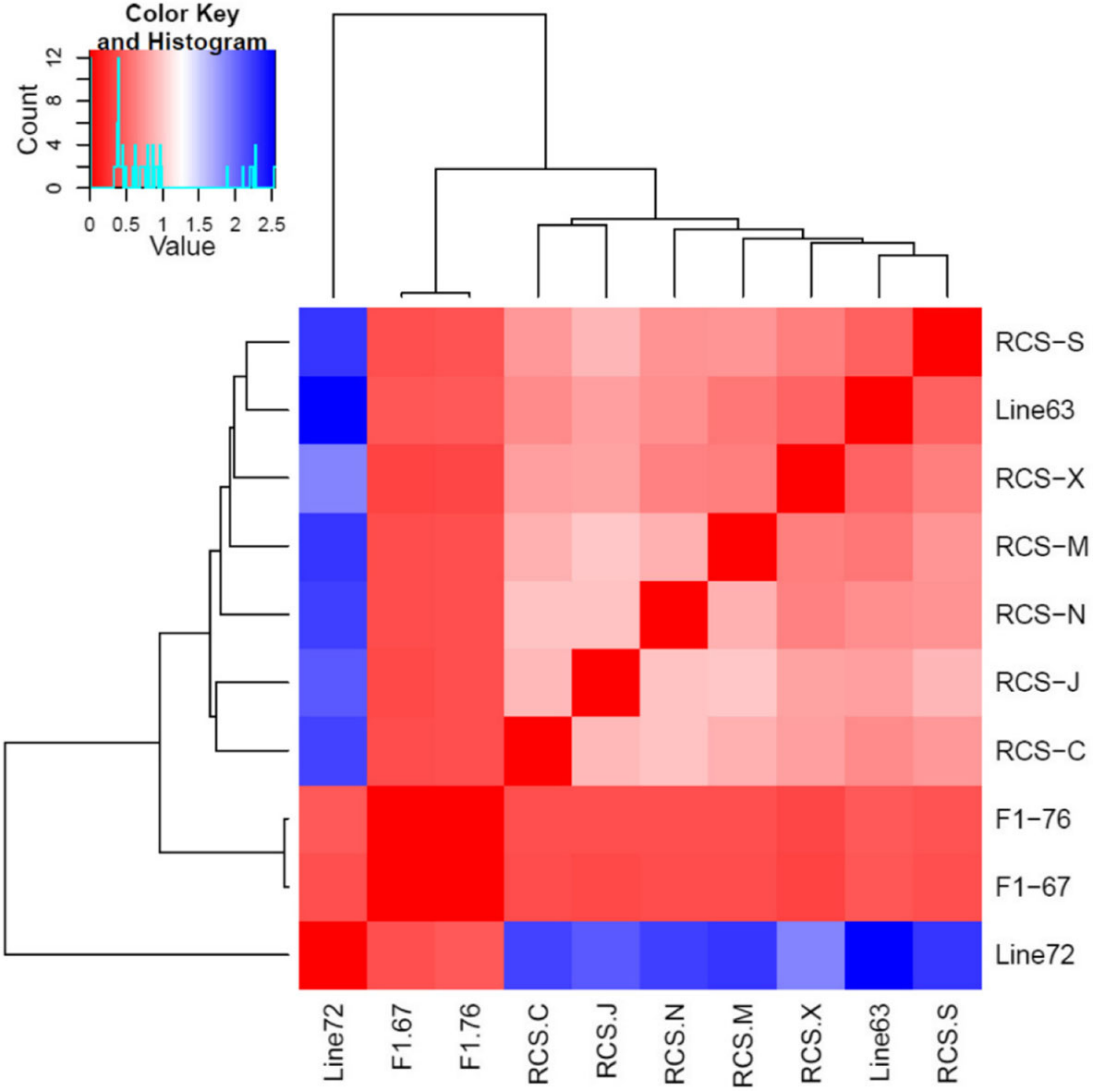
Heatmap and hierarchical clustering tree based on the Reynolds’ distance between 10 inbred lines

MDS analysis revealed the first dimension (C1) and the second dimension (C2) separated the lines into three broad non-overlapping clusters, which represent line 7_2_ (susceptibility), line 6_3_ (resistance), RCS lines and F1 (hybrid F1 generation with various level of resistance). Six F1 chickens clustered together, and they were distinguished from both susceptibility and resistance lines. We also observed 18 chickens from RCS lines were specifically clustered into resistance sub-groups distributed around line 6_3_ (Fig. 1a). Moreover, we further investigated genetic structure within 18 RCS chickens using MDS analysis, and observed J lines separated clearly from others, which indicated a differential genomic signature in J (Fig. 1b). Other lines also displayed obviously independent cluster on the MDS plots. Additionally, the results from heatmap and neighbor-joining (NJ) tree were consistent with MDS results (Fig. 2), we also found line 7_2_, next to F1 hybrid lines, were divergent from other lines.

**Fig. 3:**
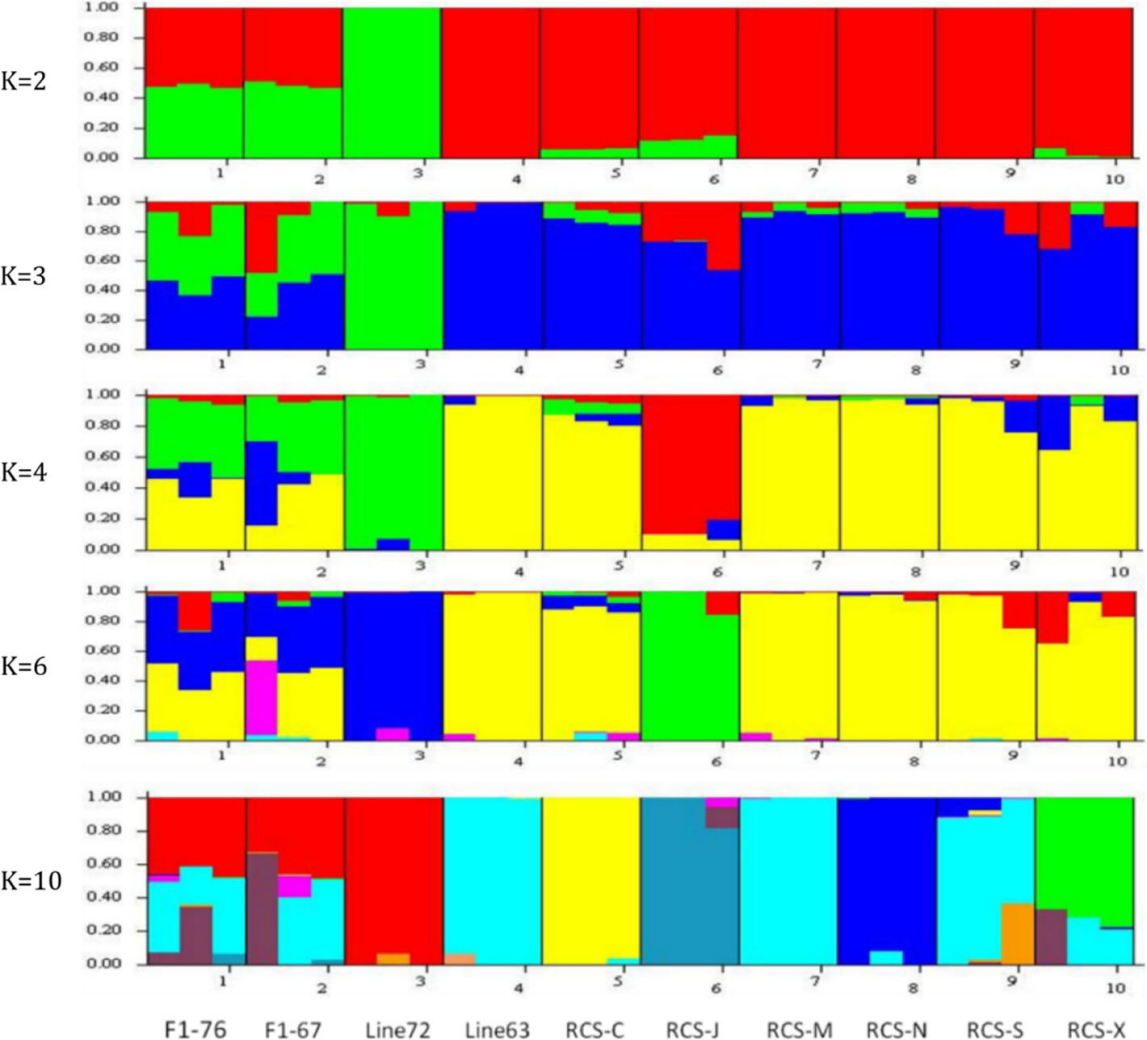
Admixture analysis using LD filtered 5,064 SNPs in all 30 chickens. Chickens are indicated by thin vertical lines partitioned into segments corresponding to the inferred membership in K = 2, K = 3, K = 4, K = 6 and K = 10 genetic clusters as indicated by the colors

**Fig. 4:**
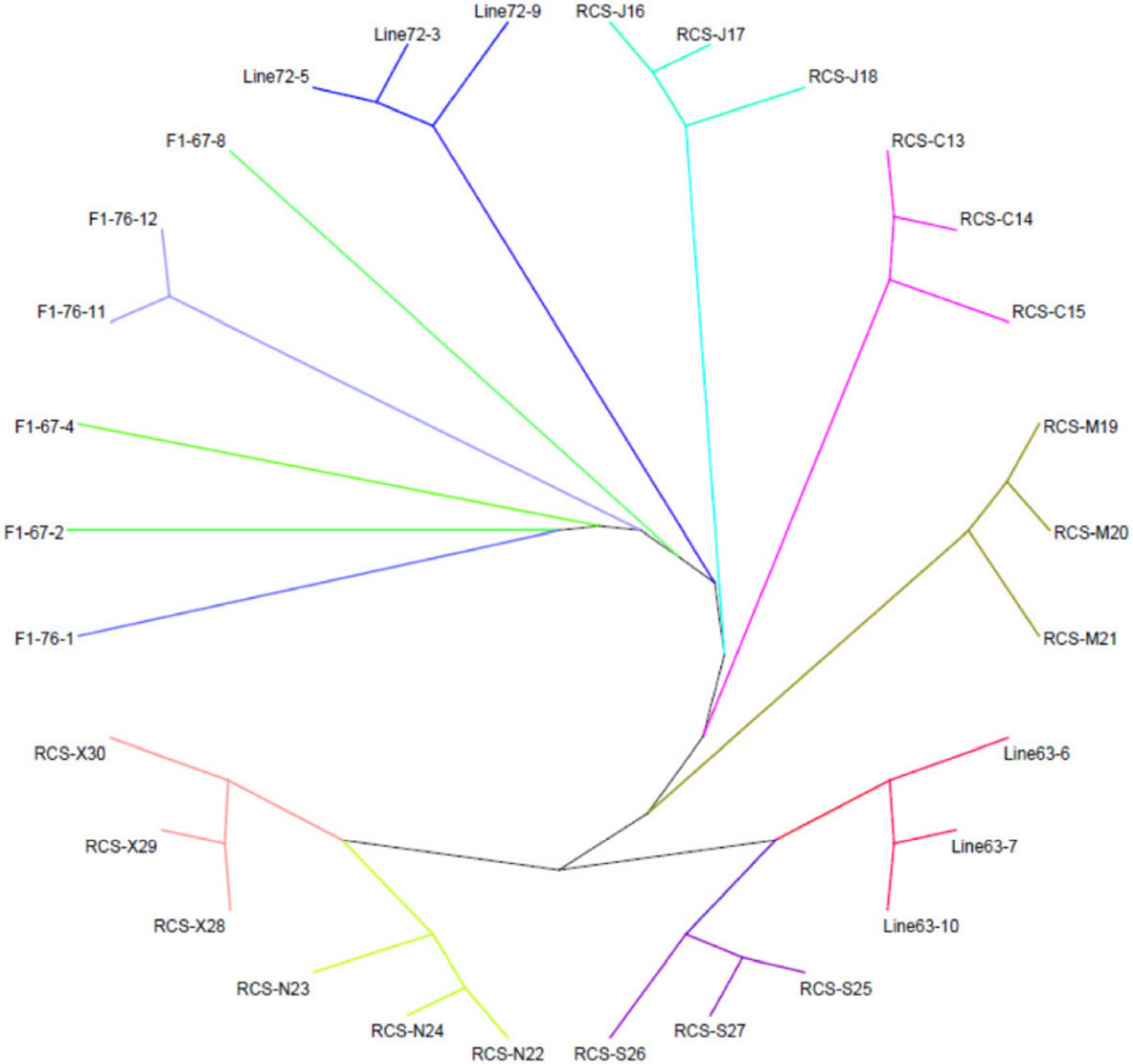
Neighbor-joining tree based on genome-wide IBD distances in 30 chickens

Then, we employed a model-based unsupervised hierarchical clustering across chickens using the program STRUCTURE. The admixture bar plot results were in well agreement with above observations with a clear separation of line 7_2_, F1, line 6_3_ and others, as shown in Fig. 3 (e.g. K = 3). In addition, as K increases to 4, J line was separated from other lines. Interestingly, when K = 10, we found line C, J, N, X were separated from line 6_3_ (indicating unique resistance lines). In addition, the neighbor-joining (NJ) tree was constructed based on IBD calculated between unambiguously separated individuals, and line 7_2_ was used as control group (Fig. 4). The results indicated detailed genetic relationships among 30 chickens as shown in Fig. 2. Three groups of inbreed lines corresponding to line 7_2_, line 6_3_, F1 could be clearly distinguished in upper, lower right and lower left, respectively. The results were also consistent with MDS and Admixture results: three individuals from different lines were clustered together at the same branches and individuals from F1 branched in an intermediary position between line 7_2_ and line 6_3_. Most importantly, all RCS lines were closely related to the line 6_3_ MD-resistant lines, which suggesting a higher influence from the genetics background of line 6_3_. In addition, we found chickens from line X, N and chickens from S, M, C, J were readily distinguishable among RCS lines, which suggested that these lines could have their unique genetic characteristics as previously reported [10].

### Genomic diverse signatures

To explore the difference of genetic polymorphism related to susceptibility and resistance during the inbreeding process, we carried out genome scan across genome using *F*_ST_ approach. After splitting the whole genome to 50 SNP windows, we obtained 10,554 raw windows. We detected 106 regions (about top 1%) with diverse signatures. To gain insight of the function enrichment of these regions, we annotated the top 106 candidate regions, and 51 genes were identified to be candidate genes for the resistance to MD. To better understand the biological processes and molecular function involved in MD genetic resistance, we performed GO term analysis using DAVID. We observed the most represented molecular function was arylamine N-acetyltransferase activity (GOTERM_MF_FAT), which included gene *PNAT10* (N-acetyltransferase, pineal gland isozyme NAT-10), *PNAT3* (N-acetyltransferase, pineal gland isozyme NAT-3), and *NAT* (N-acetyltransferase, liver isozyme) (Table 1). The most represented biological process was regulation of cell death and anti-apoptosis (GOTERM_BP_FAT). Those genes included *AKT1* (v-akt murine thymoma viral oncogene homolog 1), *CDH13* (cadherin 13, H-cadherin), *CFDP1* (craniofacial development protein 1), *API5* (apoptosis inhibitor 5), and *ING4* (inhibitor of growth family, member 4). Furthermore, KEGG_PATHWAY analysis identified *PNAT10, PNAT3, NAT* involved pathway caffeine metabolism and drug metabolism (Additional file 2: Table S1). We also detected many overlaps between the candidate regions with online chicken QTL database (http://www.animalgenome.org/cgi-bin/QTLdb/GG/index). The overlapping positions of each region and QTLs related to MD disease were listed in Additional file 3: Table S2. We observed 19 regions overlapping with known QTL regions, including 9 regions in chr1 [7, 8], 2 regions at chr2 [8, 24], 5 regions in chr5 and 1 region in chr9 [7, 8].

**Table 1:** Gene Ontology annotations of candidate genes identified using F_*ST*_ approach

GO category	GO term	Gene count	*P*-value	List of genes
GOTERM_BP_FAT	GO:0006916~anti-apoptosis	4	2.40E-04	*AKT1, CDH13, CFDP1, API5*
GOTERM_BP_FAT	GO:0042981~regulation of apoptosis	5	2.70E-03	*AKT1, CDH13, ING4, CFDP1, API5*
GOTERM_BP_FAT	GO:0043067~regulation of programmed cell death	5	2.89E-03	*AKT1, CDH13, ING4, CFDP1, API5*
GOTERM_BP_FAT	GO:0010941~regulation of cell death	5	2.94E-03	*AKT1, CDH13, ING4, CFDP1, API5*
GOTERM_BP_FAT	GO:0043066~negative regulation of apoptosis	4	3.26E-03	*AKT1, CDH13, CFDP1, API5*
GOTERM_BP_FAT	GO:0043069~negative regulation of programmed cell death	4	3.44E-03	*AKT1, CDH13, CFDP1, API5*
GOTERM_BP_FAT	GO:0060548~negative regulation of cell death	4	3.44E-03	*AKT1, CDH13, CFDP1, API5*
GOTERM_MF_FAT	GO:0004060~arylamine N-acetyltransferase activity	3	5.20E-05	*PNAT10, PNAT3, NAT*
GOTERM_MF_FAT	GO:0008080~N-acetyltransferase activity	3	2.12E-03	*PNAT10, PNAT3, NAT*
GOTERM_MF_FAT	GO:0016410~N-acyltransferase activity	3	2.71E-03	*PNAT10, PNAT3, NAT*
GOTERM_MF_FAT	GO:0016407~acetyltransferase activity	3	3.14E-03	*PNAT10, PNAT3, NAT*

### Runs of homozygosity

In this study, we found many loci had been fixed to homozygous. It is impossible to use them to explain the difference of MD resistance. However, the completed differentiations (*F*_ST_ = 1) indicates two different homozygous genotypes existed in populations. To estimate the inbred level across lines, we also calculated the inbreeding coefficient (*F*) for all 30 samples using 155,216 high quality SNPs (Additional file 4: Table S3). We found the top inbreeding coefficient (*F*) in line 6_3_, which is over 0.98. Next is line 7_2_, nearly 0.95. In contrast, the average of *F* among the RCS is 0.88 ± 0.028. Among them, the average of *F* values in RCS-S line is up to 0.94, whereas for lines N and X, *F* values are both about 0.86, which suggested there existed slight different inbred levels among inbred chicken lines.

To explore the question whether the inbred levels and homozygosity differences across the genome could contribute to the susceptibility/resistance for MD, we conducted homozygosity analysis across all 24 chickens (hybrid F1 lines were not included in ROH analysis). We obtained 2,027 ROH segments, and 1,596 of them were found in RCS lines. The genome wide results from the ROH analysis indicated obviously different ROH distributions across the genome within 24 inbred chickens. We identified 406 ROH segments in line 7_2_ and 206 ROH segments in line 6_3_. We also found ROH distribution displayed a certain degree of diversity among the six RCS lines, with the longest ROH detected in line 6_3_ and line N, about 90 Mb. However, in line 7_2_, the longest ROH was 44 Mb. The detailed summary statistics for ROH analysis, including ROH number, length and density (nSNP/kb) and SNPs within each ROH segment, was shown in Additional file 5: Table S4.

To compare the ROH distributions among 8 diverse inbred lines, we first merged ROH segments identified from all individuals for each line into nonredundent ROH regions based on genomics coordinate. We finally obtained 58, 71, 59, 64, 65, 63, 58 and 67 ROH regions in the lines 6_3_, 7_2_, C, J, M, N, S and X, respectively, while the relative more ROH regions were identified on chr1 (10 ROHs) in line 7_2_ and chr2 (8 ROHs) in line L. The most probable reason was both chromosomes cover a larger proportion of genome size as compared with other chromosomes. To facilitate the comparison of difference of ROH regions among lines, genome wide ROH regions were presented in Additional file 5: Table S4. We found some shared ROH regions, both among the RCS lines and between RCS and line 6_3_. The results indicated that ROH region caused by inbreeding may be involved in the different MD resistance. On the other hand, the unique region from line 7_2_ (susceptibility line) comparing other lines may offer some clues of genomic homozygosity region related to susceptibility.

ROH represents regions of genome where the identical haplotype is inherited from each parent. ROH regions could likely increase of recessive deleterious alleles to be co-contributed, and reducing the viability of the organism. To investigate the differential ROH distribution among diverse chickens, we further divided 24 chickens into three groups, representing parental line 6_3_ with resistance, parental susceptibility line 7_2_ group, and RCS group (including line C, J, M, N, S and X), respectively. Comparisons of ROH regions based on genomics coordinates among three groups showed that 98.6% ROH region (902.5/915.0 Mb) were shared by all three groups (Additional file 1: Figure S5).

In our study, two parental inbred line 6_3_ and line 7_2_ are MD-resistant and MD-susceptible respectively, while a Recombinant Congenic Strains (RCS) lines were developed from these two lines showing varying resistance to MD, we observed RCS lines were similar to line 63 based on genetics relation estimation in present study (Figs. 1, 2, 3 and 4). To further explore whether homozygous segments segregated with different resistance to MD diseases, all chickens with varying resistance to MD included RCS lines and line 6_3_ chickens were compared with the line 7_2_ (MD-susceptible). We obtained one region that is uniquely distributed in line 6_3_ with 513 kb length; eight regions (987 kb in size) in line 7_2_ groups, and 47 regions totaling 7,719 kb not in line 7_2_ but in shared by both RCS lines and line 6_3_ (Additional file 6: Table S5).

Subsequently, we observed 56 genes in 47 shared ROH regions, while only one gene was identified within line-specific regions in the line 6_3_. We identified eight unique regions with 13 genes in line 7_2_. Among the 56 genes identified in both line 6_3_ and RCS with different resistance, we found most of them were enriched in regulation of apoptosis and regulation of cell death, this result may indicate their potential association with the resistance of MD disease.

## Discussion

We investigated the genetic characteristics of MD resistance and susceptibility using high-density SNP array in inbred chicken lines. Genetic structure indicated differential genomic landscapes during inbreeding process in the past decades. Using genomic signature and homozygosity analysis, we identified a list of genes that may be contribute to the MD resistance or susceptibility in the inbred lines.

Previous studies have explored the genetic changes at gene expression level [10], epigenetic regulatory [25, 26], and allele specific expression [11] in the inbred chickens. To better understand the genetic mechanism underlying MD-resistance and susceptibility, we employed high density SNP arrays to investigate the genetic structure and relationship in 10 inbred lines. Using MDS analysis, we observed diverse genetic structures among them, which were highly consistent with admixture analysis and phylogenetic analysis. These chicken lines with different MD susceptibilities were separated obviously, while RCS lines were clustered together near parental line 6_3_ with similar MD-resistance. Our findings were also consistent with PCA analysis in inbred chicken using expression array [10], these results indicated similar influences on MD-resistance and susceptibility caused by SNPs and gene expression.

Although the limited samples size per line were utilized in the current study, the global F_*ST*_ approach can properly reflect the potential difference among all 30 birds. Allele frequency based method is likely to offer some important clues for searching the candidate genes involved in the MD resistance. Moreover, we found some genes related to disease and immune response. For instance, several genes were significant enriched including arylamine N-acetyltransferase activity (*AKT1*, *CDH13*, *CFDP1*, *API5*) and cell death and anti-apoptosis (*CDH13*, *ING4*, *CFDP1* and *API5*). We also detected one region located at 51.5 Mb in chromosome 5 with high selection signal (*F*_ST_ = 0.574986) that contained gene *AKT1.* AKT1 (protein kinase B, PKB) is a serine/threonine kinase that plays a critical role in regulating cell survival, insulin signaling, angiogenesis and tumor formation [27–29]. Previous studies in chicken have revealed *AKT1* as one of candidate gene for *Salmonella* response [30]. Moreover, subsequent fine-mapping of heterophil functional response to *Salmonella* in a highly advanced intercross revealed the position containing both *AKT1* and *SIVA* may work as heterophil function to explain the host-resistance properties [31]. Another gene identified in our analysis was *CDH13* was located at 15.6 Mb in chromosome 11. This gene encodes a member of the cadherin superfamily, which is hypermethylated in many types of cancer. Previous studies have showed that *CDH13* may be a promising candidate gene for Attention Deficit/Hyperactivity Disorder (*ADHD*) [32, 33] and plays a central role in the regulation of brain networks [34]. Intriguingly, we found an apoptosis inhibitory protein, coded by gene *API5* located at 20 Mb in chromosome. This apoptosis inhibitory protein prevents apoptosis after growth factor deprivation and suppresses the transcription factor E2F1-induced apoptosis, and negatively regulating acinus, a nuclearfactor involved in apoptotic DNA fragmentation. Its depletion enhances the cytotoxic action of the chemotherapeutic drugs [35].

ROH regions can increase the recessive deleterious alleles to be co-contributed, and reduce the viability of the organism. Differential ROH segments across lines may contain potential candidate genes related to MD resistance. In this study, 5 genes (*SIK, SOX1, LIG4, SIK1* and *TNFSF13B*) within ROH region were found in resistant group (line 6_3_ and RCS), and these genes have been previously reported that may be related to immunology and survival. Previous studies demonstrated that *SIK1* gene plays critical role in promoting survival of skeletal myocytes using mouse model [36] and involve with regulation of its abundance and stability for myogenesis [37]. *Sox1* as a part of the *Sox-B1* group of transcriptional regulators in neural progenitor cells is sufficient to induce neuronal lineage commitment [38]. Also, it promotes neuronal cell fate determination and differentiation by integrating multiple independent pathways. In human, the GG genotype of *LIG4* was found to be associated with higher IgE levels to *Ascaris* [39]. Also, gene *TNFSF13B*, as one of BAFF receptors, was proved to be a key survival factor during B-cell maturation [40, 41].

Interestingly, we identified one unique ROH region (at 110 Mb in chromosome 2) in line 7_2_, which contains five genes *LYN, MOS, PLAG1, TGS1* and *TMEM68*. Among them, *LYN* regulates survival and responsiveness of tumor cells by a BCR-ABL1 independent mechanism. In various hematopoietic cells, LYN has emerged as a key enzyme involved in the regulation of cell activation. In these cells, a small amount of LYN is associated with cell surface receptor proteins, including the B cell antigen receptor (BCR) [42], and CD40 [43]. *PLAG1* (pleiomorphic adenoma gene 1) was found frequently rearranged and activated in human salivary gland pleomorphic adenomas. It encodes a developmentally regulated transcription factor [44]. Ectopic overexpression of *PLAG1* has been proposed to play a crucial role in tumorigenesis of salivary gland pleomorphic adenomas. It was reported that *PLAG1* can activate the transcription of insulin-like growth factor 2 (*IGF2*), functioning as a proto-oncogene [45]. *PLAG1* is a proto-oncogene whose overexpression is a crucial oncogenic event in salivary gland pleomorphic adenomas (PA), and in carcinoma ex pleomorphic adenoma (CA-ex-PA). Our results provided some important insights for understanding the molecular mechanisms of MD resistance. Moreover, we identified gene *USP15* (ubiquitin-specific protease 15) in line 6_3_, which may indicate its potential role for MD resistance. USP15 have been reported that have an essential role for regulation of caspase-3 during Paclitaxel-induced apoptosis [46] and TGF-β pathway in human studies [47].

## Conclusions

We investigated the genomic characteristics of inbred chicken lines using high density SNP array by integrating genomic signature, ROH analysis. Our findings revealed that several candidate genes including *AKT1*, *CDH13*, *CFDP*, *API5*, and *USP15* for MD susceptibility. Future studies with large sample size and fine mapping of the genetic variants using powerful association statistics would elucidate the complex genetic mechanisms of resistance/susceptibility to MD.

## Additional files

Additional file 1: **Figure S1.** Histogram plot of MD incidence (%) rate. MD resistance in chickens is generally evaluated with MD incidence (induced gross tumors by MDV) and survival day post MDV challenge, and the extent of resistance is dependent of the virulence of challenge viruses and other factors. **Figure S2.** Barplot show the call rate for 30 chickens on Affymetrix chicken SNP genotyping array. X axis represent the call rate values, and Y axis represents the chicken individuals. **Figure S3.** Frequency distribution in autosomes for a total of 527,021 SNPs on the Affymetrix chicken SNP genotyping array. **Figure S4.** Frequency distribution in autosomes for 155,216 SNPs on the Affymetrix chicken SNP genotyping array. **Figure S5.** Comparison of ROH regions among three groups, line 6_3_, line 7_2_, lines RCS (C, L, M, N, S, X). The overlap length of ROH was indicated in kb. (DOCX 35 kb)

Additional file 2: **Table S1.** GO analysis for 51 genes in 106 identified candidate regions. (XLSX 20 kb)

Additional file 3: **Table S2.** The overlapping positions for 106 identified candidate regions with QTLs related to MD disease. (XLSX 12 kb)

Additional file 4: **Table S3.** The inbreeding coefficient (*F*) for all 30 samples using 155,216 high quality SNPs. (XLSX 11 kb)

Additional file 5: **Table S4.** Summary statistics and ROH distributions among 8 diverse inbred lines including lines 6_3_, 7_2_, C, J, M, N, S and X. (XLSX 31 kb)

Additional file 6: **Table S5.** Unique and shared homozygous segments across all chickens included RCS lines and line 6_3_ chickens were compared with the line 72. (XLSX 13 kb)

## Abbreviations

ADOL: Avian disease and oncology laboratory
BC: Reciprocal backcross
IBD: Identity-by-descent
IBS: Identical by state
LD: Linkage disequilibrium
MD: Marek’s disease
MDS: Multidimensional scaling
MDV: Marek’s disease virus
NJ: Neighbor-joining
QTL: Quantitative trait loci
RCS: Recombinant congenic strains
ROH: Runs of homozygosity
