# Supplementary material for: Genetic assessment of inbred chicken lines indicates genomic signatures of resistance to Marek’s disease
Source: J Anim Sci Biotechnol. 2018 Sep 13;9:65. doi: 10.1186/s40104-018-0281-x (PMC6136188; doi:10.1186/s40104-018-0281-x)
Supplement: Supplementary file 1 — Figure S1. Histogram plot of MD incidence (%) rate. MD resistance in chickens is generally evaluated with MD incidence (induced gross tumors by MDV) and survival day post MDV challenge, and the extent of resistance is dependent of the virulence of challenge viruses and other factors. Figure S2. Barplot show the call rate for 30 chickens on Affymetrix chicken SNP genotyping array. X axis represent the call rate values, and Y axis represents the chicken individuals. Figure S3. Frequency distribution in autosomes for a total of 527,021 SNPs on the Affymetrix chicken SNP genotyping array. Figure S4. Frequency distribution in autosomes for 155,216 SNPs on the Affymetrix chicken SNP genotyping array. Figure S5. Comparison of ROH regions among three groups, line 63, line 72, lines RCS (C, L, M, N, S, X). The overlap length of ROH was indicated in kb. (DOCX 35 kb) [file 40104_2018_281_MOESM1_ESM.docx]

Figure S1. Histogram plot of MD incidence (%) rate. MD resistance in chickens is generally evaluated with MD incidence (induced gross tumors by MDV) and survival days post MDV challenge. The MD incidence data presented in Figure S1 were totally based on numbers of chickens that developed tumors post MDV challenge. The calculation was done as the ratio of number of birds with tumors/total number of birds challenged within each of the lines multiplied by 100.

Figure S2. Barplot of call rate for 30 chickens Affymetrix Axiom® Genome-Wide Chicken Genotyping, X axis represent the call rate values, and Y axis represents the chicken individuals.

Figure S3.Frequency distribution in auto chromosome for the total SNPs for the Affymetrix Axiom® Genome-Wide Chicken Genotyping Array(600K)

SNP count 527021.

Figure S4. Frequency distribution in auto chromosome for the total SNPs for the Affymetrix Axiom® Genome-Wide Chicken Genotyping Array(600K)

SNP count 155216 SNPs.

Figure S5 Comparison of ROH Region among three groups, Line _63_, Line _72_, Line RCS (C, L, M, N, S, X). The overlap length of ROH were indicated in Kb.
